# Epidemiological profile of measles in Niger: analysis of measles case-based surveillance data from 2010 to 2019

**DOI:** 10.11604/pamj.2022.43.18.33443

**Published:** 2022-09-12

**Authors:** Habibatou Amadou Idé, Pauline Kiswendsida Yanogo, Djibril Barry, Ousmane Boua Togola, Eric Adehossi, Nicolas Meda

**Affiliations:** 1 Burkina Field Epidemiology and Laboratory Training Program (BFELTP), Université Joseph Ki Zerbo, Ouagadougou, Burkina Faso,; 2 Medical School, University of Ouaga 1 Joseph Ki Zerbo, Ouagadougou, Burkina Faso,; 3 Medical School, University Abdoul Moumouni, Niamey, Niger

**Keywords:** Measles, surveillance, database, Niger

## Abstract

Measles is a rapidly growing disease in the world with 869,770 cases and 207,500 deaths recorded in 2019. Niger continues to record epidemic outbreaks despite the actions taken. This study aims to analyze the national database from 2010 to 2019 to characterize the epidemiology of measles in Niger. This is a descriptive retrospective study. Our sample is exhaustive of suspected and positive measles cases from the database of the department of surveillance and response to epidemics for 10 years. Data extraction and analysis was done using Epi Info 7.2.3.1 software. In our study we found n=11,784 suspected measles cases notified from 2010 to 2019 with 37.2% of positive cases (IgM+). All regions are concerned. The female/male sex ratio was 1.1. The 1-to-5-year age group was the most representative (44.44%); 28.3% received at least one dose of vaccine; 62.22% lived in urban areas. The number of deaths was 225 (1.9%). The proportion of samples received at the laboratory within 3 days is 70.38%. The baseline analysis allowed us to find that all regions recorded cases and deaths with a low vaccination rate of 28.3%. Improved response and immunization strategies are recommended.

## Introduction

Measles, one of the most contagious diseases in the world, is caused by a virus, which belongs to the paramyxovirus family. Although generally benign, it remains one of the major causes of death in young children, yet there is a safe and effective vaccine [1]. The World Health Organization (WHO) recommends 95% vaccination coverage with two doses of measles vaccine in every country and in all communities to protect populations from the disease. The coverage rate for the first dose of vaccine has stagnated at a global scale for over a decade, now between 84% and 85% and that for the second dose is gradually increasing but is only 71% in 2020 [2,3].

Globally, WHO estimated that less than one in 10 cases were reported, with differences between regions. In 2019, there were 869,770 cases of measles and the number of deaths increased by almost 50%; 207,500 deaths were the highest level since 1996, with the increase occurring in all WHO regions [4,5]. In Africa, measles epidemics have been reported in several countries. In 2019, cases and deaths were recorded in the Democratic Republic of Congo (more than 310,000 cases and 6,000 deaths), in the Central African Republic (5,724 cases including 83 deaths), in Ethiopia (over 12,000 cases), and in Chad (26,623 cases including 259 deaths). Infections are not only a sign of insufficient measles vaccination coverage, but an indicator that vital health services do not always reach the populations most at risk [2].

In Niger, measles is a notifiable disease. In a non-epidemic situation, each suspected case should be filled with a blood sample between the 4^th^ and 28^th^ day after the onset of the rash. In a non-epidemic situation, each suspected case should be filled with a blood sample between the 4^th^ and 28^th^ day after the onset of the rash. In the event of an epidemic, the occurrence of five suspected cases in a month in the integrated health centers (IHC) should suggest a possible epidemic and trigger the conduct of an outbreak investigation at the identified health structures to determine the actual extent and causes of occurrence [6].

Measles outbreaks continue to be recorded in several districts, sometimes two weeks after the vaccination campaign in Niger. In 2019, in Niger the number of suspected cases was 2,568 with 431 confirmed cases. This number was significantly high compared to the 1,445 suspected cases and 397 positive cases recorded in 2018 [7]. This was after several actions had been undertaken including the establishment of case-by-case measles surveillance, the strengthening of the expanded routine vaccination program with the introduction of a catch-up dose of the measles vaccine in children under one-year-old, strengthening communication activities. It was necessary to conduct a database analysis to characterize the epidemiology of measles in Niger and improve the surveillance system.

## Methods

**The study framework:** Niger is a landlocked country in West Africa. It has a surface area is 1,266,491 km^2^ and extends between 11°37 and 24°33 of the north latitude and 0°06 and 16° of the east longitude, with Niamey as its capital. Niger is bordered to the north by Algeria and Libya, to the east by Chad, in the south by Nigeria and Benin and to the west by Burkina Faso and Mali. According to the last general population and housing census of 2012, the population of Niger was 17,138,707 inhabitants with a projection of 22,314,743 inhabitants in 2019, that is an average density in 2019 of 17.62 inhabitants/km^2^. Children under 15 years old represent 51.32% of the population, an estimated fertility rate of 7.6 children per woman, and an estimated life expectancy at birth of 59 years for men and 60 years for women (EDSN-MICS IV 2012). More than 80% of the Nigerien population live in rural areas of agriculture and livestock. Variations in rainfall, archaism in cultivation and livestock practices, and poor organization of markets push the inhabitants of villages to settle in periurban areas. The conditions are then precarious and contribute to the spread of infectious pathologies. According to the WHO, 63% of deaths in Niger in 2016 were due to largely preventable causes: infectious diseases, prenatal care, management of insufficient childbirth, and malnutrition [8].

The health system has been organized into a three-level health pyramid since it joined the Lusaka Declaration in 1985 and that of Bamako in 1987. Niger has adopted a 2011-2015 multi-year comprehensive plan as part of the 2011-2015 Health Development Plan (HDP), in accordance with the objective set by the African Region of WHO to reduce measles mortality by 98% by 2015 [6]. Among the objectives are: 1) achieve at least 98% reduction in measles-related mortality in 2012; 2) achieve at least 95% routine vaccination coverage for measles vaccine nationally and 80% from all regions; 3) achieve at least 95% coverage for supplementary vaccination activities against measles in all regions; 4) at least 80% of districts must report at least one suspected case of measles with specimen of blood each year. On the other hand, Niger is committed to eliminating by 2020 indigenous measles as a major public health problem in application of its health development policy and in accordance with the resolution of the 130^th^ Executive Committee of WHO and the orientations of the 61^st^ meeting of the WHO/AFRO Regional Committee [6].

**Figure 1:**
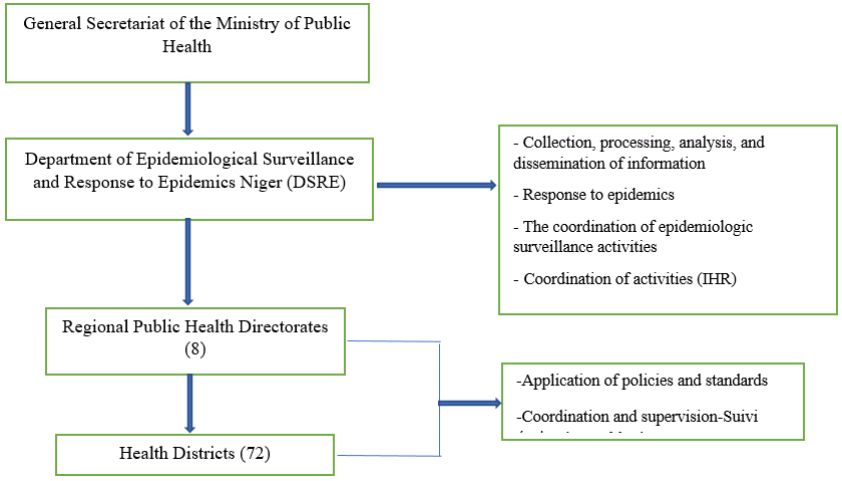
Niger Department of Surveillance and Response to Epidemics organizational chart (IHR: international health regulations)

**Field of study:** the Department of Surveillance and Response to Epidemics (DSRE) based in Niamey was created by Decree No. 2011-21/PRN/MSP of October 26, 2011. It is located at the central level of the health pyramid. Its function is to coordinate the epidemiological surveillance activities of the health sector; prepare the response to epidemics, disseminate, and maintain data relating to the epidemiological surveillance of diseases, maternal deaths in the country, and ensure continuous training of health personnel. It is made up of a secretariat, a resource management service, an epidemiologic surveillance division, an epidemic response division and a communication division (Figure 1).

**Description of the study population:** a descriptive retrospective study was carried out at the DSRE on measles cases notified in all health structures in Niger as part of case-by-case surveillance. The study period spanned ten (10) years, from 2010 to 2019. The WHO case definition criteria were used to define measles cases: 1) suspected cases: anyone with a generalized maculopapular rash and fever, plus one of the following signs: cough, coryza (runny nose), conjunctivitis (red eyes) or anyone for whom a clinician suspects measles; 2) confirmed cases: this concerns any suspected case confirmed by the laboratory (positive measles IgM) or having an epidemiological link with confirmed cases or an epidemic outbreak.

**Laboratory examinations:** centrifuged whole blood samples from all districts of Niger were sent to the National Reference Laboratory (LNR), an auxiliary of the Niamey National Hospital. Upon receipt, the samples contained in the cryotubes were dated and labelled with an identification number (e.g. NIG-DOS-BOB-20-001) indicating the country (NIG), the region of origin (e.g. DOS), the health district (e.g. BOB), the year (20), and the order of receipt (e.g. 001). Thereafter, samples are split into 2 parts, one part stored at -20°C and the other part processed directly. The remainders of the treated parts were stored at -20°C and a quarter used quality monitoring, 10% of collected samples were sent to the Institute Pasteur in Abidjan for external quality control. Every year, WHO sends 10 measles and rubella samples for treatment with good scores at the LNR. According to the WHO diagnostic algorithm, if a test result is negative (IgM-), a follow-up test is done for anti-rubella IgM; if the measles result is unknown, the sample is retested.

**Statistical data analysis:** we used Epi Info software version 7.2.3.1 to extract data from the 2010-2019 measles surveillance database and perform statistical analyzes. Descriptive statistical analysis was performed with results presented as frequencies and means. In addition, data for some variables were presented as tables and graphs.

**Ethical considerations:** our protocol on analysis was submitted to the program coordination of the Burkina Faso Field Epidemiology and Laboratory Training Program (BFELTP) and authorization to proceed with the data analysis was obtained at the level of the Department of Epidemiological Surveillance and Response to Epidemics Niger (000125/P/AS/DGPS/DSRE of August 12, 2020). Confidentiality was assured and maintained. The identification number assigned to the patient that is considered and data collected was kept anonymous.

## Results

A total of 11,754 suspected measles cases were reported in Niger over a 10-year-period (2010-2019). Missing data on all the variables was 76 or 0.64%.

**Sociodemographic characteristics and vaccination status of the study population:** of the 11,754 subjects, 6,240 (53%) were women, that is a female/male sex ratio of 1.1. The median age of suspected cases was 4 years with an age range from 0 to 99 years. The age group 1 to 5 years represents 44.44% followed by the age group 6 to 14 years (23.34%). A total of 3,324 (28.3%) people received at least one dose of measles vaccine. Among the suspected cases 7,308 (62.22%) lived in rural areas (Table 1). The number of deaths was 225 yielding a case fatality rate of 1.9%.

**Table 1:** socio-demographic characteristics and vaccination status of suspected measles cases in Niger from 2010 to 2019

Characteristics	Frequency (N)	Percentage (%)
**Age (N=11754)**		
Less than 1 year	1408	11.98
1 - 5 years	5224	44.44
6 - 14 years	2743	23.34
15 years and older	2379	20.24
**Sex (N=11754)**		
Male	5514	47.00
Female	6240	53.00
**Geographic classification (N=11744)**		
Urban	7308	62.22
Rural	4436	37.78
**Immunizations (N=11754)**		
Vaccinated	3324	28.30
Not vaccinated	3874	33.00
Unknown	4556	38.70
N: number		

**Figure 2:**
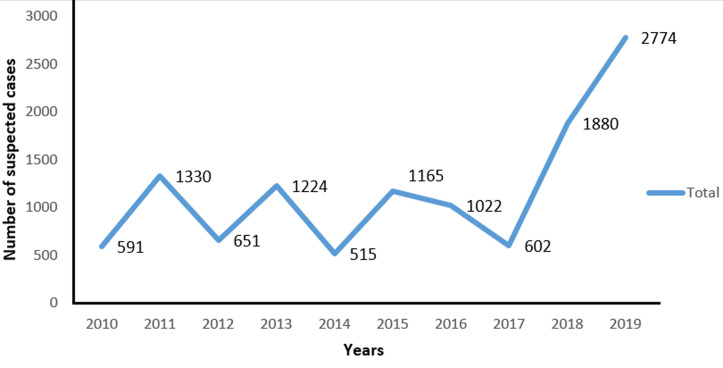
development of the number of suspected measles cases per year in Niger from 2010 - 2019

**Development of suspected measles cases from 2010-2019:** the number of suspected cases has seen sharp peaks and dips over the 10 years with a significant increase in cases in 2019 (Figure 2) which joined the global trend in the same year with high rates recorded.

**Figure 3:**
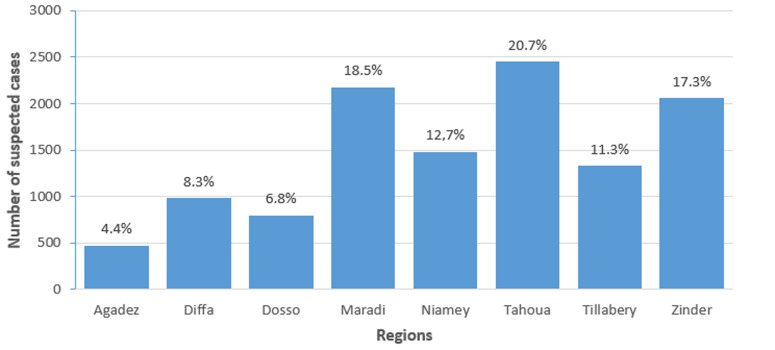
distribution of the number of suspected measles cases by region in Niger from 2010 - 2019

**Distribution of suspected cases by region:** the suspected measles cases came from rural areas in 62.22% of patients. The distribution of cases by region shows that the were 2,433 cases (20.7%) reported in the Tahoua Region, 2,177 cases (18.5%) reported in Maradi, and 2,040 cases (17.3%) reported the Zinder Region (Figure 3).

**Epidemiological aspects of laboratory-confirmed cases:** out of a total of 11,754 suspected cases, IgM measles serology was performed in 7,598 at the national reference laboratory with 57.6% (IgM+) and 4380 confirmed patients, i.e. a rate of 37.2% compared to all suspected cases. The median age of confirmed cases was 3 years. The number of confirmed cases (1937) was higher in the 1 to 5-year age group representing 44.22% and 15 years and over with 918 cases or 20.96%. A total of 2292 (52.33%) cases were male with a female/male sex ratio of 0.9. The Tahoua Region recorded the highest number of confirmed cases (978) at 22.32%, followed by Maradi (835) at 19.06%, and Zinder (798) with 18.21% of cases during the study period.

**Figure 4:**
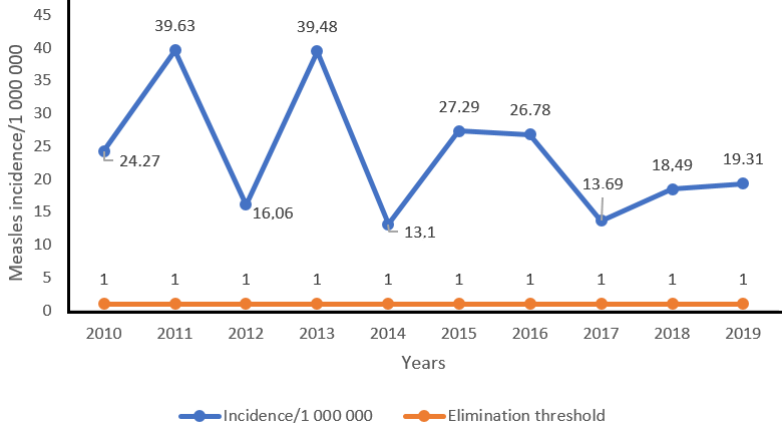
development of the annual incidence of measles in Niger from 2010 to 2019

**Incidence of measles by year:** the incidence of measles has developed two sharp peaks in 2011 and 2013, that is, 39.63 and 39.58 cases per 1,000,000 inhabitants, respectively (Figure 4).

**Measles surveillance performance indicators:** the analysis of the performance indicators showed that 2 in 4 indicators were achieved, namely “the proportion of samples received at the laboratory in good condition” and the “proportion of samples taken within 30 days of rash onset”. In contrast, “the proportion of districts that reported at least one suspected case of measles with blood sampling in the year”, “the proportion of samples received at the laboratory within 3 days after collection” and “promptness of feedback on the results of serology by the laboratory” (Table 2).

**Table 2:** results of measles performance indicators in Niger

Performance indicators	Results	Indicators
Proportion of districts having notified at least 1 suspected case of measles with blood sample in the year	66.14%	≥ 80%
Proportion of samples received at the laboratory within 3 days after collection	70.38%	≥ 80%
Proportion of samples received at the laboratory in good condition	94.26%	≥ 80%
Proportion of samples taken within 30 days of the onset of the rash	98,7%	≥ 80%
Promptness of feedback on serology results by the national reference laboratory, less than 7 days after receipt of samples	56.89%	≥ 80%

## Discussion

This study was made possible thanks to data from the measles surveillance centralized at the DSRE level. It revealed certain shortcomings, in particular the lack of information on certain important variables (clinical signs) with a completeness of 93.6%. On the other hand, the reliability of the data did not pose a problem as the heads of regional and district offices made regular visits to the field to verify them. In addition, all samples were sent for confirmation at the national reference laboratory, an auxiliary of the National Hospital of Niamey. The study also revealed several important findings. In addition to the quality of the data collected by the surveillance system, the populations still at risk of contracting measles in Niger, and how the national measles case treatment system operated during the period studied.

**Sociodemographic characteristics and vaccination status of the population studied:** this study showed that the median age of suspected cases was 4 years with range of 0 to 99 years and was below the mean age of 6.3 years (14 days to 66 years) found by Farra *et al*. [9]. The most affected age groups were children aged 1 to 5 (44.44%). Similar observations were observed in other African countries where the most affected groups were the 1 to 4 years old (36.87%) in Niger [6], in Mali the 0 to 5 years (24%) [10], in Ethiopia 1 to 4 years (31.3%) [11], children under 5 (67.4%) in Senegal [12], and children aged 9 months to 5 years in Uganda [13].

We can therefore see that the age 0-to-5-year age group was the most affected and corresponds to the period when children are more likely to contract a disease because their immunity is being built. Measles outbreaks occur when people who are not immune to the virus become infected and transmit the disease to unvaccinated or insufficiently vaccinated populations. Infections are not only a sign of insufficient measles immunization coverage, but are also a known marker, an indicator that vital health services do not always reach the populations most at risk [2]. Out of the total of 11,754 suspected cases, 6,240 (53%) were women, yielding a female/male sex ratio of 1.1. The result was confirmed by Alkassoum *et al*. in Niger (53%) [6]. Farra *et al*. had found (51.7%) [9] while Coulibaly *et al*. [10] had found a male sex predominant at 56.25% as well as Palamara *et al*. in Italy (54%) [14]. The place of origin was more urban (62.22%) than rural and a similar result was reported by Coulibaly *et al*. (64.58%) [10], the majority of cases were urban according to Alkassoum *et al*. [6].

In our sample, 71.4% of the subjects were not vaccinated, these results are lower than those noted in studies by Seck *et al*. 88.5% [12], Alkassoum *et al*. 93.1% [6], and Palarama *et al*. in Italy (95%) [14]. This means that many children skip vaccination campaigns and are the weakest link in the chain of measles transmission.

**Epidemiological aspects of laboratory-confirmed cases:** during the measles surveillance from 2010 to 2019, 11,754 suspected cases were reported and the percentage of confirmed out of all suspected cases was 37.26%. This rate is higher compared to 31.3%, 24.6%, 16.0%, 6% and 6.7% respectively found by other studies [9-11,15,16]. Among the confirmed cases, the age group 1 to 5 years represented 44.22% compared to 51.4% of the 1 to 4 year old age group in Nigeria, Fatiregun *et al*. [15] Both in terms of suspected and confirmed cases, the regions of Tahoua (22.32%), Maradi (19.06%), and Zinder (18.21%) were the most affected.

**Measles surveillance performance indicators:** a third of districts reported at least one suspected case with blood sampling during the year were (66.44%), the proportion of samples received at the laboratory within 3 days was 70.38%, and the promptness of the feedback information on the results of serology by the laboratory was 56.89%. An Alkassoum *et al*. [6] study on the same objectives observed frequencies of 43%, 40.17%, and 89.77%, respectively. The proportion of samples received at the laboratory in good condition was 94.26%.

## Conclusion

In our study, it was observed that despite the efforts made to eliminate measles in Niger, it remains a public health problem because epidemic outbreaks are still recorded. Only 37.2% of reported cases were laboratory confirmed, children aged 1 to 5 years were the most affected, without a big difference between the sexes. Not all target groups have reached the recommended vaccination status (28.3%). Therefore, it is important to identify populations in areas with low coverage and at higher risk of epidemics, to strengthen the routine vaccination program, to improve the response and vaccination strategies throughout the country and encourage research.

### What is known about this topic

- Measles rose sharply around the world in 2019;
- Measles remains one of the major causes of death in young children;
- This disease can be almost completely prevented with two doses of a safe and effective vaccine.

### What this study adds

- The most affected age groups were children aged 1-5 years;
- In our sample, 71.4% of subjects were not vaccinated;
- Three out of five measles surveillance performance indicators are not met.
